# Inhibition of *L. monocytogenes* Biofilm Formation by the Amidase Domain of the Phage vB_LmoS_293 Endolysin

**DOI:** 10.3390/v11080722

**Published:** 2019-08-06

**Authors:** Vincenzo Pennone, Marta Sanz-Gaitero, Paula O’Connor, Aidan Coffey, Kieran Jordan, Mark J. van Raaij, Olivia McAuliffe

**Affiliations:** 1 Teagasc Food Research Center, Moorepark, Fermoy, Co. Cork P61 C996, Ireland; 2 Centro Nacional de Biotecnología (CNB-CSIC), 28049 Madrid, Spain; 3 Cork Institute of Technology, Bishopstown, Cork T12 P928, Ireland

**Keywords:** listeriophage, endolysin, amidase

## Abstract

*Listeria monocytogenes* is a ubiquitous Gram-positive bacterium that is a major concern for food business operators because of its pathogenicity and ability to form biofilms in food production environments. Bacteriophages (phages) have been evaluated as biocontrol agents for *L. monocytogenes* in a number of studies and, indeed, certain phages have been approved for use as anti-listerial agents in food processing environments (ListShield and PhageGuard Listex). Endolysins are proteins produced by phages in the host cell. They cleave the peptidoglycan cell wall, thus allowing release of progeny phage into the environment. In this study, the amidase domain of the phage vB_LmoS_293 endolysin (293-amidase) was cloned and expressed in *Escherichia. coli (E. coli)*. Muralytic activity at different concentrations, pH and temperature values, lytic spectrum and activity against biofilms was determined for the purified 293-amidase protein. The results showed activity on autoclaved cells at three different temperatures (20 °C, 37 °C and 50 °C), with a wider specificity (*L. monocytogenes* 473 and 3099, a serotype 4b and serogroup 1/2b-3b-7, respectively) compared to the phage itself, which targets only *L. monocytogenes* serotypes 4b and 4e. The protein also inhibits biofilm formation on abiotic surfaces. These results show the potential of using recombinant antimicrobial proteins against pathogens in the food production environment.

## 1. Introduction

In recent years, a number of studies have focused on finding novel biotechnological approaches to prevent or remove *L. monocytogenes* biofilms [1,2,3]. Promising results have been obtained with the utilisation of phages for *L. monocytogenes* removal [2,4,5,6]. Phages are bacterial viruses that can infect host cells with high specificity. For *L. monocytogenes* biocontrol in the food industry, two products containing phages are currently commercially available: Listex P100 (www.phageguard.com) and ListShield (www.intralytix.com). Listex P100 contains the phage P100, a wide spectrum virulent listeriophage, which has been proven to remove *L. monocytogenes* from foods and biofilms on food processing environment surfaces [4,5,7,8,9]. ListShield is a cocktail of six listeriophages with demonstrated efficacy against a wide spectrum of *L. monocytogenes* strains, both in foods and on biofilms [2,10,11,12,13]. Both phage preparations have been approved by the US Food and Drug Administration (FDA) to be used during food production and are under revision by the European Food Safety Authority [6].

From the perspective of using whole phages as biocontrol agents, virulent phages have been regarded as much better options when compared to temperate phages [14]. The main reason for this is the possibility that bacterial cells might not be killed by the temperate phages because of their exclusion by immunity mechanisms. Another reason is the ability of temperate phages to potentially transfer unwanted genetic material to target hosts through lysogenic conversion [15]. Furthermore, phage resistance can be developed in the bacteria [16]. For these reasons, the use of different phages and phage-derived proteins are being investigated, including the use of phage endolysins [17].

Endolysins, or lysins, are enzymes encoded by the phage genome involved in the release of phage progeny from the host cell. Their mode of action is through the cleavage of the peptidoglycan of the host cell wall [18]. They usually act in conjunction with other phage-encoded proteins, known as holins, a diverse group of small proteins that are involved in the formation of holes in the inner membrane of the host cell, thus allowing the endolysin access to the peptidoglycan [19]. Based on the presence of certain catalytic domains that determine which peptidoglycan bond is cleaved, phage endolysins can be divided in five groups: muramidases, lytic transglycosylases, glucosaminidases, amidases and endopeptidases [17]. Using recombinant endolysins as antimicrobial agents rather than whole phages limits the possibility of development of bacterial resistance to the endolysin. Endolysins recognize and cleave highly immutable targets in the cell wall—the peptidoglycan bonds. A mutation at this level would most likely compromise the fitness of the bacterial cells [20]. The peptidoglycan is located extracellularly, which reduces the number of mechanisms of resistance to endolysins when applied from without. Many resistance mechanisms, such as the reduction of membrane permeability, active efflux or inactivation by cytoplasmic enzymes, target antimicrobials acting inside the cell [21,22]. Some endolysins possess several catalytic domains that hydrolyse different bonds in the peptidoglycan, which further reduce the chance of resistance development [23]. In addition to this, endolysins can be used in combination with other endolysins or antimicrobials to further reduce the development of resistance, showing synergistic effects in some cases [24,25,26]. Studies have already shown reduction of *L. monocytogenes* cell numbers through the exogenous application of endolysins. For example, PlyP100, the endolysin encoded by the listeriophage P100, has shown anti-listerial activity when applied to drained curd during the production of fresh cheese, with only 0.5 log cfu/g reduction, but preventing the growth of *L. monocytogenes* for more than 3 weeks [27]. The same endolysin, combined with nisin, was applied during the production of queso fresco, showing a reduction in *L. monocytogenes* numbers to under the detection limit. By contrast, the application of nisin alone showed very little anti-listerial activity at the time of application, with consecutive regrowth of *L. monocytogenes* to levels comparable to the untreated controls [28]. The endolysin LysZ5 of the phage FWLLm3 has shown more than 5 log_10_ CFU mL^−1^ reduction of *L. monocytogenes*, *L. innocua* and *L. welshimeri*, without affecting *Staphylococcus. aureus* or *Enterococcus. faecalis* in soya milk [29].

Endolysins against Gram-positive bacteria are usually formed by two or more domains, generally including a catalytic domain and a cell wall binding domain. Further studies have shown the possibility of using the catalytic domains of endolysins rather than the full-length protein as biocontrol agents. In some cases, this resulted in a display of increased activity compared to the full length endolysin, as shown by the activity of the cysteine- and histidine-dependent amidohydrolase/peptidase (CHAP) domain of the staphylococcal phage endolysin LysK against strains of *S. aureus* [30]. In that study, the CHAP_K_ domain of LysK showed a broader lytic spectrum compared to the full endolysin and lytic activity at different temperatures, pH and concentrations [30]. Recently, novel technologies have been developed to increase the antimicrobial effect of endolysins, such as the production of beads of polyhydroxyalkanoate (PHA) bionanoparticles (BNPs) displaying on the surface the protein of interest [31], or microencapsulation, for example, into Poly(N-isopropylacrylamide) (PNIPAM) nanoparticles, which allowed the release of CHAP_k_ and lysostaphin when reaching a temperature of 37 °C [32].

Phage vB_LmoS_293 is a temperate member of the Siphoviridae family, which infects strains of *L. monocytogenes* of the serotypes 4b and 4e [33]. Whole genome sequencing of this phage by our group revealed its temperate nature [33]. The issues associated with employing temperate phages for the purposes of biocontrol, described earlier in this paragraph, led to the pursuit of the endolysin of this phage as a potential biocontrol agent. The endolysin of phage vB_LmoS_293, an N-acetylmuramoyl-l-alanine amidase, was chosen for further study. The aim of this work was to produce a recombinant amidase, the catalytic domain of the vB_LmoS_293 endolysin, referred to as 293-amidase, and characterisation of its antimicrobial activity.

## 2. Materials and Methods

### 2.1. Bacterial Strains and Culture Conditions

The *L. monocytogenes* strains used for this study are detailed in Table 1. The listed strains were isolated from two different projects and, depending on the project requirements, the serotype or the serogroup were determined via agglutination test or PCR, respectively. All the strains were stored at −20 °C and grown in Brain Heart Infusion (BHI, Oxoid Ltd., Basingstoke, UK) broth at 37 °C. For the cloning and expression of the 293-amidase, *E. coli* TOP10 and *E. coli* BL21(DE3) were used, respectively (Table 1). Transformed *E. coli* strains were stored at −80 °C and grown in Lysogeny Broth (LB) containing 50 µg/mL kanamycin at 37 °C.

### 2.2. Bioinformatics Analysis

The listeriophage vB_LmoS_293 genome was previously annotated and deposited in the GeneBank database with the Accession Number KP399678.1 [34]. The sequence of the open reading frame (ORF) coding for the phage endolysin (GenBank accession number AJE28090.1) was analysed with the Basic Local Alignment Search Tool (BLAST) against the Conserved Domains Database (CDD, [35]) for identification of the amidase domain (293-amidase).

### 2.3. Amidase Cloning

Cloning of the 293-amidase was performed using the TOPO-TA cloning kit (Thermo Fisher Scientific, Waltham, MA, USA), which includes the pCR^®^2.1-TOPO^®^ vector, following the manufacturer’s instructions. The pCri-8a was chosen as the expression vector for this study [36]. Phage DNA was extracted with the phenol chloroform-isoamyl alcohol as described in Reference [34]. For cloning, the polymerase chain reaction (PCR) and topoisomerase reaction were performed using the TOPO-TA cloning kit (Thermo Fisher Scientific, USA) following the manufacturer’s instructions. The primers designed for the PCR reaction were: Amidase_Fw_NcoI CGCGCCATGGCCATGTCAGTACTACAATATAATTATATCAATAAAAATCAATTT and Amidase_Rv_HindIII CGCGCAAGCTTGCACCTTTCAATTTTGCATTG. The pCR^®^2.1-TOPO^®^-293-amidase vector was transformed into chemically competent *E. coli* Top10 cells (Thermo Fisher Scientific, Waltham, MA, USA), following the manufacturer’s instructions. The transformed cells were then plated on Lysogeny Broth (LB, Oxoid Ltd., UK) agar containing 50 µg/mL kanamycin and 40 µL of 40 mg/mL X-gal (both supplied by Sigma, Arklow, Ireland) and incubated for 16 h at 37 °C. White colonies were selected and screened for the presence of the insert with a colony PCR and grown for 16 h at 37 °C in LB broth with 50 µg/mL kanamycin, shaking at 250 rpm. Plasmid DNA was extracted from each of the selected colonies using the QIAprep Spin mini-prep kit (Qiagen, Germany).

The 293-amidase was excised from the pCR^®^2.1-TOPO^®^ vector by double digestion with the endonucleases NcoI and HindIII (Thermo Fisher Scientific, USA), the pCri-8a plasmid was linearized by double digestion with the same endonucleases and a ligation reaction (T4 DNA Ligase, New England Biolabs, UK) was performed for 16 h at room temperature. Chemically competent *E. coli* TOP10 cells were transformed with the ligation product, according to the manufacturer’s instructions. The transformed cells were plated on LB agar containing 50 µg/mL kanamycin and incubated 16 h at 37 °C. Single colonies were grown in LB broth with 50 µg/mL kanamycin, shaking at 250 rpm and the plasmids were recovered with a mini-prep kit and sequenced by Eurofins GATC, Germany. Chemically competent *E. coli* BL21(DE3) (Thermo Fisher Scientific, USA) were transformed with the plasmids containing the 293-amidase gene, following the manufacturer’s instructions and plated on LB agar Petri dishes containing 50 µg/mL kanamycin and incubated for 16 h at 37 °C.

Sequences were analysed with the CLC Main Workbench 8 (https://www.qiagenbioinformatics.com/products/clc-main-workbench/).

### 2.4. Protein Expression and Purification

One colony of *E. coli* BL21(DE3) transformed with pCri-8a-293-amidase was incubated for 16 h in 10 mL of Terrific Broth (TB, 23.6 g/L yeast extract, 11.8 g/L tryptone, 9.4 g/L K_2_HPO_4_, 2.2 g/L KH_2_PO_4_, 8 mL/L glycerol) with 50 µg/mL kanamycin (TB-K) at 37 °C, shaking at 250 rpm. A 3 L flask containing 990 mL of TB-K was inoculated with 10 mL of a 16 h aerobic culture of *E. coli* BL21(DE3) transformed with pCri-8a-293-amidase and incubated at 37 °C, shaking at 100 rpm. When the culture reached 0.6 OD_600_, the cells were chilled on ice for 10 min and then induced by adding 1 mL isopropyl β-D-1-thiogalactopyranoside (IPTG, Sigma, Ireland) 1 M (final concentration: 1 mM) and incubated for a further 16 h at 16 °C. After incubation, the cells were chilled on ice for 10 min and harvested by centrifugation at 2000× *g* for 30 min at 4 °C (Sorvall™ RC 6 Plus, Fisher Scientific, Canada). The cell pellet was resuspended in 10 mL of lysis buffer (50 mM Tris, 300 mM NaCl, 10% glycerol) and frozen until lysis was performed. The thawed cell pellet was lysed by sonication (MSE Soniprep 150, Fisher Scientific, UK) with 5× medium frequency impulses for 10 s followed by 10 s of rest on ice. The lysate was centrifuged in a top bench centrifuge at maximum speed for 30 min at 4 °C and the clarified supernatant was filtered through a 0.22 μm syringe filter (Sarstedt, Germany). Subsequently, the filtrate was incubated with 2 mL of Immobilised Metal Affinity Chromatography (IMAC) resin containing nitrilotriacetic acid charged with Ni^2+^ (Ni-NTA) (Bio-Rad, USA) for 1 h on an orbital shaking platform. The mix was transferred to a gravity chromatography column (Econo-Pac, Bio-Rad, USA) and affinity chromatography purification was performed according to the manufacturer, using a running buffer composed of 5 mM NaH_2_PO_4_, 30 mM Na_2_HPO_4_, 300 mM NaCl, 45 mM imidazole. After three washing steps with running buffer, the 293-amidase was eluted in four fractions containing 145, 245, 345, and 545 mM imidazole, respectively. The fractions containing the purified 293-amidase were pooled together and dialyzed for 16 h at 4 °C, using a dialysis membrane with a 10 kDa cut-off 10 kDa (VWR International Ltd., Ireland) in 1 L of phosphate buffer (NaH_2_PO_4_ 5 mM, Na_2_HPO_4_ 30 mM, NaCl 300 mM) to remove the imidazole. The purified protein was stored at −20 °C in 0.5 mL aliquots. The concentration of the 293-amidase was estimated using the Pierce BCA protein assay (Thermo Fisher Scientific, Waltham, MA, USA), following the microtiter plate protocol from the manufacturer guidelines.

### 2.5. In-Gel Digestion and Matrix-Assisted-Laser-Desorption/Ionization Time-of-Flight (MALDI-TOF)

Mass spectrometry (MS) grade Pierce Trypsin Protease (Rockford, IL, USA) was dissolved in 50 mM acetic acid to a final concentration of 10 ng/μL. Three microlitres of trypsin solution was added to 10 µL of 293-amidase 150 μg/mL for digestion. Ten microlitres of 0.5 mM ammonium bicarbonate was added and the solution digested in a heating block at 37 °C for 3.5 h. Mass spectrometry was performed on the digested sample with an Axima TOF2 MALDI-TOF mass spectrometer (Shimadzu Biotech, Manchester, UK). A 0.5 μL aliquot of matrix solution (α-cyano 4-hydroxy cinnamic acid, 10 mg/mL in 50% acetonitrile-0.1% (*v*/*v*) trifluoroacetic acid) was deposited onto the target and left for 5 s before being removed. The residual solution was allowed to air-dry and 0.5 μL of the sample solution was deposited onto the pre-coated sample spot. A 0.5 μL aliquot of matrix solution was added to the deposited sample and allowed to air dry. The sample was subsequently analysed in positive-ion reflectron mode. Protein identification was carried out via peptide mass fingerprinting (PMF) using the Mascot search engine (http://www.matrixscience.com). The monoisotopic, positive ion data +/− 0.25 Da were searched using the following parameters: NCBInr database or Swiss Prot, taxonomy all entries, trypsin digest with one missed cleavage.

### 2.6. Determination of the Activity of 293-Amidase against Live Cells of *L. monocytogenes*

*Listeria monocytogenes* 473, the strain used to propagate vB_LmoS_293, was used to test the lytic activity of 293-amidase against live cells. For this purpose, the Clinical and Laboratory Standards Institute (CLSI) protocol for antimicrobial dilution and disk susceptibility test protocol was used [37]. Briefly, *L. monocytogenes* 473 was grown in BHI broth at 37 °C for 16 h, the cells were harvested by centrifugation, resuspended in sterile saline solution to a concentration of 0.5 McFarland standard, and stored at −20 °C. A sterile cotton swab was used to spread the bacterial solution on the surface of a BHI agar plate and disks for susceptibility testing (Oxoid Ltd., Basingstoke, UK) impregnated with 293-amidase at the concentrations of 150, 100, 50, 25, 12.5, and 6.25 µg/mL were applied on its surface. Lysozyme (20 mg/mL, Sigma, Arklow, Ireland) was used as positive control and sterile phosphate buffer as negative control. The plates were incubated at 37 °C for 16 h and checked for zones of inhibition.

### 2.7. Determination of 293-Amidase Muralytic Activity at Different Concentrations

To determine the muralytic activity, the 293-amidase was tested against *L. monocytogenes* 473, the strain used to propagate the phage vB_LmoS_293. For the preparation of cells, *L. monocytogenes* 473 was grown 16 h in 100 mL of BHI broth and the cells were subsequently autoclaved at 121 °C for 15 min (Astell Scientific, UK). The cells were harvested by centrifugation at 2000× *g* for 15 min, washed with 10 mL sterile water and aliquots frozen until required. The LB agar plates were overlaid with 5 mL of LB top agar (0.7% agar) containing 1 g of *L. monocytogenes* 473 autoclaved cells. To determine the muralytic activity, 10 μL of 293-amidase at increasing concentrations (5 µg/mL, 10 µg/mL, 20 µg/mL, 40 µg/mL, 75 µg/mL and 150 µg/mL) was spotted on a lawn of *L. monocytogenes* 473, dried in a laminar flow cabinet, incubated at 37 °C and examined every 30 min for the presence of lysis.

The muralytic activity was determined in parallel in microtiter plates (Corning Inc., Kennebunk, ME, USA). The autoclaved *L. monocytogenes* 473 cells were resuspended in sterile phosphate buffer (NaH_2_PO_4_ 5 mM, Na_2_HPO_4_ 30 mM, NaCl 300 mM) to an OD_620_ of 1.0. A volume of 100 μL of the cell suspension was aliquoted onto a microtiter plate well. The muralytic activity was tested as follows: 100 μL of purified 293-amidase was added to the cells to a final concentration of 5 µg/mL, 10 µg/mL, 20 µg/mL, 40 µg/mL, and 75 µg/mL, respectively, and the reduction in turbidity at OD_620_ was recorded with a microplate reader (Synergy HT, Biotek Instruments, USA) at the time 0, 20, 45, 75, 105 min. The data were normalized with the controls with the equation: ((OD_Control_ − OD_293-amidase_) × 100/OD_initial_) and the percentage of reduction was shown on scatter plots.

### 2.8. Influence of Temperature on 293-Amidase Activity

To check the optimal temperature for activity, 1 μg of 293-amidase was spotted on a lawn of *L. monocytogenes* 473, prepared as described previously, incubated at 25 °C, 37 °C and 50 °C and examined every 30 min for lysis. Additionally, in microtiter plates, 100 μL of autoclaved *L. monocytogenes* 473 cells, resuspended in phosphate buffer to an OD_620_ of 1.0 was added to 100 μL of 293-amidase at a final concentration of 40 μg/mL and incubated at 25 °C, 37 °C and 50 °C. As negative control, 100 μL of autoclaved *L. monocytogenes* 473 cells, resuspended in phosphate buffer to an OD_620_ of 1.0 was added to 100 μL of phosphate buffer. The reduction in turbidity was recorded with a microplate reader at the times 0, 30, 90, 180 min and 24 h. The 293-amidase reduction were normalized as described before and shown on scatter plots.

### 2.9. Specificity of 293-Amidase

To assess the lytic activity against different bacterial strains, overnight (16 h) cultures in BHI were prepared for each of the strains listed in Table 1. The cultures were autoclaved and the pellets were resuspended as described previously. The LB plates were overlaid with 5 mL of LB top-agar 0.7%, containing 1 mL of each autoclaved strain, spotted with 1 μg of 293-amidase and incubated at 37 °C, checking every 30 min for the presence of lysis. The appearance of a clearing zone was considered a confirmation of lytic activity against the strains tested.

The specificity of 293-amidase was tested also as turbidity reduction at OD_620_. The autoclaved strains listed in Table 1 were resuspended in phosphate buffer to an OD_620_ of 1.0. In a microtiter plate, 100 μL of resuspended cells was added to 100 μL of 293-amidase at a final concentration of 40 µg/mL. The reduction in turbidity at OD_620_ was measured with a microplate reader every 10 min at an incubation temperature of 37 °C.

### 2.10. Influence of pH on 293-Amidase Activity

Buffers at pH 4 (Na-citrate 20 mM, pH 4), pH 8 (Tris-HCl 20 mM, pH 8) and pH 10 (boric acid 20 mM, pH 10) were used to resuspend autoclaved cells of *L. monocytogenes* strain 473 to assess the influence of pH on the amidase activity. The cells were resuspended to an OD_620_ of 1.0 and, in a microtiter plate, 100 μL of cell suspension was mixed with 100 µL of 293-amidase at a final concentration of 40 µg/mL and the reduction in turbidity at OD_620_ was recorded every 10 min by a microplate reader with incubation temperature of 37 °C.

### 2.11. Efficacy of 293-Amidase against *L. monocytogenes* Biofilm

The 293-amidase antibiofilm activity was tested on polystyrene microtiter plates and stainless-steel coupons. To assess the efficacy of the amidase to prevent adhesion of *L. monocytogenes* to an abiotic surface, 10 μL of an overnight (16 h) culture of *L. monocytogenes* 473 was added to 90 μL of BHI broth in a microtiter plate well. In the control wells, 100 μL of phosphate buffer was added, while in the treatment wells, 100 μL of 293-amidase at a final concentration of 75 µg/mL was added to the cell suspensions. The microplate was incubated for 5 days at 20 °C and the biofilms were stained with crystal violet [1]. The absorbance at 595 nm was recorded with a microplate reader.

To test the efficacy of the 293-amidase on *L. monocytogenes* biofilm removal, 20 μL of an overnight (16 h) culture was added to 180 μL of BHI broth in a microtiter plate well. After four days of incubation at 20 °C, the wells were washed with phosphate buffer saline (PBS, Sigma, Ireland) to remove unattached cells and 100 μL of phosphate buffer was added to the control wells, while 100 μL of 293-amidase at a final concentration of 75 µg/mL was added to the treatment wells. After 24 h of incubation at 20 °C, the microplate was stained with crystal violet and the results recorded as stated previously.

### 2.12. Fluorescence Microscopy on Stainless-Steel Coupons

Stainless-steel (ss) coupons (10 by 15 mm, type AISI-304, no. 2b, finish, 3 mm thick) were prepared as described in Reference [1]. Briefly, the coupons were treated with acetone, washed with a detergent, rinsed thoroughly with water and autoclaved at 121 °C for 15 min. Each coupon was placed in a well of a square Petri dish with 25 compartments (Thermo Fisher Scientific, USA) and tested for the efficacy of the 293-amidase to prevent adhesion and for biofilm removal. To test the anti-adhesion activity, to each well containing a coupon, 500 μL of BHI broth plus 10 μL of a 16 h culture of *L. monocytogenes* 473 were added. To the control and the treatment, 500 μL of phosphate buffer or 75 μg of 293-amidase in 500 μL phosphate buffer were added, respectively. After 5 days of incubation at 20 °C, the coupons were washed with PBS to remove unattached cells and stained with live/dead fluorescent dye (Thermo Fisher Scientific, Waltham, MA, USA) containing Propidium Iodide (excitation/emission 535/617 nm) and Syto9 (excitation/emission 485/498 nm), following the manufacturer’s instructions. Biofilms were visualised with a Leica DMi8 fluorescent microscope and images processed with the LAS X software (Leica, Wetzlar, Germany).

To test the 293-amidase biofilm removal activity, 1 mL of BHI with 10 μL of a 16 h culture of *L. monocytogenes* 473 was added to each well containing a coupon. Biofilms were grown for 4 days at 20 °C and then the coupons were washed with PBS and 500 μL of phosphate buffer or 75 μg of 293-amidase in 500 μL phosphate buffer were added respectively to the control and the treatment. After 24 h of incubation at 20 °C, the coupons were washed again with PBS, stained with live/dead fluorescent dye and observed as described previously.

### 2.13. Statistical Analysis

Turbidity reduction assays were performed in independent triplicates and the data obtained were analysed with the Student’s *t*-test.

## 3. Results

### 3.1. Cloning, Expression and Purification of the 293-Amidase

Bioinformatic analysis of the phage vB_LmoS_293 genome suggested that nucleotides 19966–20916 (951 bp) of ORF 25 encoded the 316 amino acid endolysin and that the protein belongs to the N-acetylmuramoyl-L-alanine amidase CwlA family (COG5632), with residues 23–157 belonging to the amidase 2 family (pfam01510). Residues 179–316 had a sequence identity of 78% with residues 189–326 of *Listeria* endolysin PlyPsa (PDB code 1XOV), corresponding to the cell wall binding domain of this enzyme, with no associated catalytic activity [38]. A gene fragment encoding residues 1 to 178 of the putative vB_LmoS_293 phage endolysin, i.e., the predicted amidase domain plus flanking N-terminal and C-terminal residues, was PCR-amplified from the genome of vB_LmoS_293 and cloned into the expression vector pCri-8a, using the NcoI and HindIII restriction sites [36]. The DNA sequence analysis of the expression plasmid confirmed the correct insertion, although a single silent point mutation was observed (Supplementary Materials Figure S1B). The predicted length of the expressed protein was 219 amino acids (including the N-terminal His6 purification tag), with a predicted mass of 25 kDa and an isoelectric point of 9.3. In Supplementary Materials Figure S2, an SDS-PAGE gel shows the over-expression of a protein between 25 kDa and 35 kDa. This protein was confirmed as the 293-amidase by peptide mass fingerprinting (Supplementary Materials Figure S3). The concentration of the purified 293-amidase was 150 μg/mL.

### 3.2. Activity of 293-Amidase against *L. monocytogenes*

The disk diffusion assay showed no inhibition against any of the live strains by any of the 293-amidase dilutions tested, while inhibition was shown by the lysozyme used as positive control.

### 3.3. Determination of Muralytic Activity of 293-Amidase

**Figure 1:**
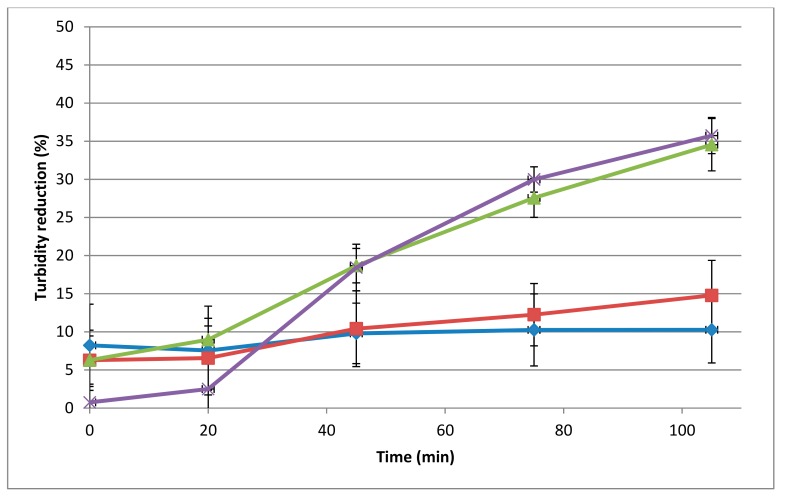
Dose-dependent muralytic activity of the 293-amidase against autoclaved *L. monocytogenes* 473 cells. Lytic activity (assays in microtiter plates) is represented as the percentage of turbidity reduction operated by the 293-amidase at different concentrations. Different colours are different concentrations: blue, 10 µg/mL; red, 20 µg/mL; green, 40 µg/mL; purple, 75 µg/mL. Each data point is the average of triplicates and the standard deviations are indicated as error bars.

The spot-on-lawn and microtiter assays performed on autoclaved *L. monocytogenes* 473 cells showed muralytic activity starting from a concentration of 10 μg/mL. In the microtiter plate assays, this concentration gave a 10% reduction in 105 min incubation (Figure 1, *p* < 0.05), while a 34.5% and 35.7% reduction in autoclaved *L. monocytogenes* 473 in 105 min incubation was observed at 40 μg/mL and 75 μg/mL, respectively (*p* < 0.05).

### 3.4. Influence of pH and Temperature on Activity of 293-Amidase

The 293-amidase showed activity at different pH conditions. There was no significant difference (*p* > 0.05) in the activity of the 293-amidase at the pH values tested: 38%, 41% and 45% at pH values 4, 8 and 10, respectively.

**Figure 2:**
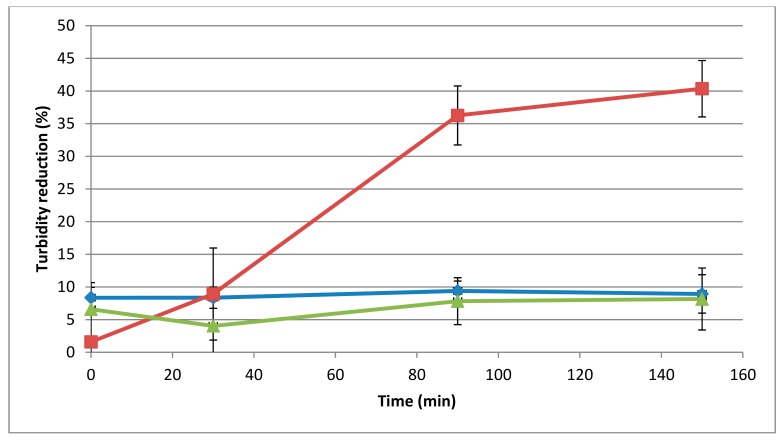
Influence of temperature on the lytic activity of the 293-amidase (40 μg/mL) against autoclaved *L. monocytogenes* 473. Lytic activity datasets are represented as in Figure 1. Different line colours are different temperatures: blue, 25 °C; red, 37 °C; green, 50 °C.

The temperature influenced the activity of the 293-amidase (Figure 2), showing 40% of turbidity reduction in microtiter plates after 150 min of incubation, and visible lysis on a lawn of *L. monocytogenes* 473 after 30 min of incubation at 37 °C. By contrast, at 25 °C and 50 °C, the turbidity reduction after 150 min of incubation was 8.9% and 8.1%, respectively, and on the spot-on-lawn assay, the lysis was shown only after 3 h of incubation.

### 3.5. Specificity of 293-Amidase

**Figure 3:**
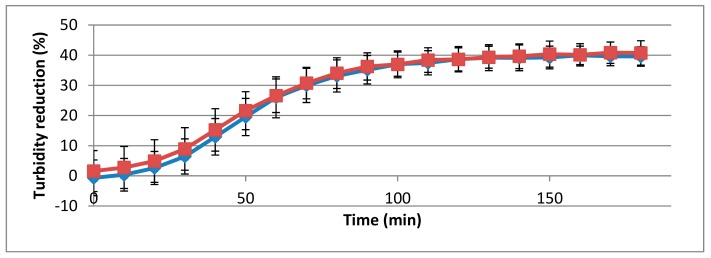
Muralytic activity of the 293-amidase domain (40 μg/mL) against *L. monocytogenes* strains 473 (red) and 3099 (blue). Lytic activity datasets are represented as in Figure 1.

Of the strains tested, described in Table 1, the 293-amidase showed muralytic activity only in the presence of *L. monocytogenes* 473 (serotype 4b) and 3099 (serogroup 1/2b-3b-7), with a statistically significant difference between the controls and treatments (*p* < 0.05, Figure 3).

### 3.6. Biofilm Inhibition and Removal

**Figure 4:**
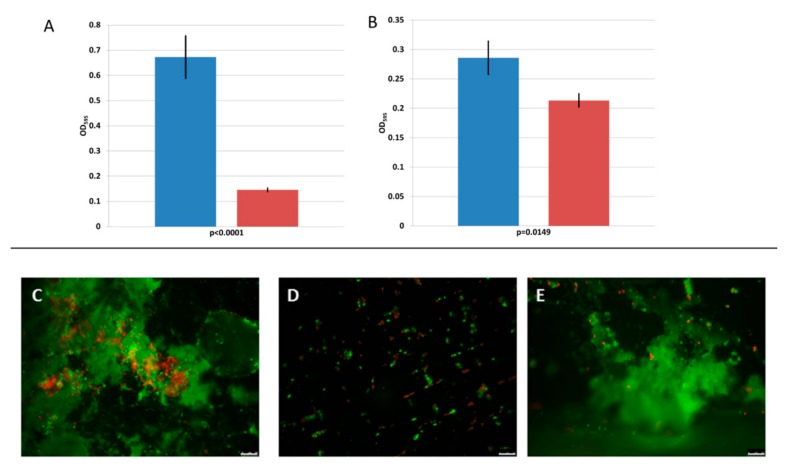
Biofilm prevention (**A**) and biofilm removal ability (**B**) of the 293-amidase (75 μg/mL) against *L. monocytogenes* 473 in microtiter plates assays. The blue bars represent the controls (untreated biofilm) and the red bars the treatments. Values are the average of triplicates and the standard deviation is represented as error bars. The *p*-values are shown below the horizontal axes. (**C**–**E**) The images obtained from the stainless-steel coupons assays. (**C**) *L. monocytogenes* 473 biofilm after 4 days of incubation at 20 °C. (**D**) Co-inoculation of *L. monocytogenes* 473 and 293-amidase (150 μg/mL). (**E**) The application of 293-amidase (150 μg/mL) on a 4 day old biofilm. The red-stained cells were damaged or dead, while the green stained cells were alive. The scale bars at the bottom-right in (**C**–**E**) are 10 µm.

The tests performed on biofilm inhibition and removal are summarised in Figure 4. Incubation of 75 µg of 293-amidase with *L. monocytogenes* 473 at 20 °C resulted in a 78% reduction (*p* < 0.0001) in the formation of biofilm on polystyrene microplates tested after 5 days of incubation (Figure 4A). The 293-amidase applied to a 4 day old biofilm resulted in a 25% reduction on the pre-formed biofilm (*p* < 0.05, Figure 4B).

On stainless steel coupons, the 293-amidase inhibited the formation of a biofilm, while no evidence of removal was observed when the protein was applied on a 4 day old biofilm (Figure 4C–E). Live and damaged cells, stained with the Live/Dead fluorescent dye, were observed in both the controls and treated biofilms.

## 4. Discussion

In food safety, the use of phage-derived proteins as antimicrobials and for biofilm removal is currently being investigated [19,33]. Using only the catalytic domains rather than the full-length endolysin can facilitate the production of the recombinant protein, by increasing their stability and solubility and, in some cases, can improve their activity [39,40]. The results described in this work have shown that the 293-amidase is able to degrade the peptidoglycan of autoclaved *L. monocytogenes* 473 cells, starting from a concentration of 10 μg/mL. However, on live cells, no inhibition was shown. It is common practice to freeze the substrate cells before use, to make them susceptible to phage enzymes, as described in previous studies (e.g., [41]), but the damage inflicted by the 293-amidase to live cell walls is apparently not sufficient to overcome their ability to recover. The results shown in this study, although showing no lethal effect on live cells under the experimental conditions attempted, highlighted the possibility of using temperate bacteriophage DNA to produce recombinant endolysins or, in this case, single domains, for biocontrol purposes.

For this reason, the 293-amidase was tested against autoclaved cells. The specificity range of 293-amidase is broader than that of the phage itself, because of the amidase activity recorded against a 1/2b-3b-7 strain. Further characterisation of the 293-amidase showed a reduction in turbidity at all the pH values tested, but there was no significant difference in the values tested between pH 4 and 8. It is possible that the different buffer components used influenced this, but as there was no difference in the activity at the pH values tested, further studies were not undertaken. In previous studies, improved activity was observed at pH 8–9 by other endolysins [42,43].

The antimicrobial activity of the 293-amidase showed the potential for preventing biofilm formation. When 293-amidase was co-inoculated with *L. monocytogenes* 473, biofilm formation was inhibited, both in polystyrene microtiter plates and on stainless-steel coupons. In contrast, when applied to a four-day old biofilm, the 293-amidase showed a reduction of 25% in polystyrene plates, while on stainless-steel coupons no differences were observed between the control and treatment. Possibly, the differences were minimal and could not be visualised, or the biofilm formed on stainless-steel coupons was thicker than the biofilm formed on polystyrene plates and was less affected by the application of the 293-amidase, although high-binding microwell plates were used in this study. This would contradict past studies, where differences in *L. monocytogenes* biofilm formation ability related to the surfaces tested were shown, with a stronger biofilm formed on polystyrene surfaces than the one formed on stainless-steel coupons [44,45]. It is likely that the damage induced by the 293-amidase to the peptidoglycan of the bacterial cells prevents adhesion on abiotic surfaces. In this way, the formation of the biofilm, both on polystyrene microtiter plates and stainless-steel coupons, was inhibited. Prevention of biofilm formation would lead to many benefits, in particular to the food production industry. The choice of the abiotic surfaces used in this study for biofilm formation was driven by the necessity of having general screening data on 293-amidase potential biofilm inhibition, data produced by the polystyrene microtiter plates, and the necessity of testing a material commonly used in the food production environment. Stainless steel is, indeed, commonly used, for example, for tables, platforms and cutters in many branches of food production [45]. To date, anti-listerial activity of endolysins has been mainly focused on in vitro experiments on planktonic cells or only a few food matrices [46]. Only the endolysin PlyLM, identified by Simmons et al. (2012) [47] in the genome of a *L. monocytogenes* 4b strain, has been previously tested against *L. monocytogenes* biofilms [47]. In that case, a monolayer biofilm was grown for 24 h at 37 °C and PlyLM reduced the biofilm by 20%, while completely removing the biofilm when combined with a protease. Further investigations might be required to assess if the same activity is registered at lower temperatures, such as 20 °C, as tested in this study, which more accurately reflects the food production environment.

The antibiofilm activity observed in this study highlights a potential application of 293-amidase in the food production industry, where a major problem is represented by the cross contamination of *L. monocytogenes* from the processing environment to the final product [48,49,50,51]. Furthermore, the association between biofilm formation and sanitiser resistance in *L. monocytogenes* [52] highlights the importance of the anti-biofilm properties shown by the purified 293-amidase. However, optimisation of the production process is required. It needs to be highlighted that microtiter plate assays have some limitations, especially the possible evaporation of the buffer, with condensation on the lid, after long periods of incubation, which increases the standard deviation of the measurements. Furthermore, the starting cell suspension was set at 1 OD_620_ with a standard spectrophotometer cuvette, but after the application of cell suspension and treatments to the microtiter plate wells, the starting OD_620_ was between 0.2 and 0.4, probably due to the different measurement system used by the standard spectrophotometer (with horizontal light path) and the microtiter plate reader (with vertical light path).

The combination of lysins and other treatments, in some cases, has shown to improve their effectiveness in pathogen control in the food production by improving hurdle technology, for example the combination of PlyP100 with nisin in fresh cheese [28]. In that study, nisin alone had a poor anti-listerial effect on queso fresco cheese, while PlyP100 had a stronger anti-listerial activity. It was demonstrated that the combination of the two treatments improved the *L. monocytogenes* removal from queso fresco, with occurrence below the detection limit after 4 weeks of incubation [28].

Studies on the application of endolysins in the food production environment are still limited. The 293-amidase potential for biofilm prevention on abiotic surfaces highlights the need of further studies to show the effect of this novel treatment in vivo.

## 5. Conclusions

In this study, the 293-amidase showed anti-listerial activity on autoclaved cells and this activity was characterised for specificity, pH, temperature, and concentration. The results on biofilms grown on microtiter plates showed a potential preventive activity against *L. monocytogenes* biofilm and a reduction in a four-day old biofilm. The 293-amidase was not active against live cells; however, its muralytic activity can still be considered for prevention of *L. monocytogenes* biofilm formation. Further studies are required to optimise the process of 293-amidase production and its possible application in the food processing environment, where tools to prevent biofilm formation are needed.

## Figures and Tables

**Table 1:** Strains used in this study.

Strain ID	Serotype/Serogroup	Source
*L. monocytogenes* 473 *	4b	Dairy industry
*L. monocytogenes* 777 *	1/2c	Dairy industry
*L. monocytogenes* 2075 *	4b-4d-4e	Mushroom industry
*L. monocytogenes* 2081 *	1/2a-3a	Mushroom industry
*L. monocytogenes* 3099 *	1/2b-3b-7	Mushroom industry
*L. innocua* *		Dairy industry
*L. welshimeri* *		Dairy industry
*L. ivanovii* *		Dairy industry
*L. seeligeri* *		Mushroom industry
*Bacillus. cereus* *		Dairy industry
*Staphylococcus aureus* S24 *		
*E. coli* Top 10		Thermo Fisher Scientific
*E. coli* BL21 (DE3)		Thermo Fisher Scientific

* These strains were provided by the collection of Teagasc, Moorepark, Fermoy, Co. Cork.

## Supplementary Materials

Supplementary materials can be found at https://www.mdpi.com/1999-4915/11/8/722/s1.
