# Supplementary material for: Inhibition of L. monocytogenes Biofilm Formation by the Amidase Domain of the Phage vB_LmoS_293 Endolysin
Source: Viruses. 2019 Aug 6;11(8):722. doi: 10.3390/v11080722 (PMC6723838; doi:10.3390/v11080722)
Supplement: Supplementary file 1 [file viruses-11-00722-s001.zip › viruses-482321 final supplementary/Figure S1.pdf]

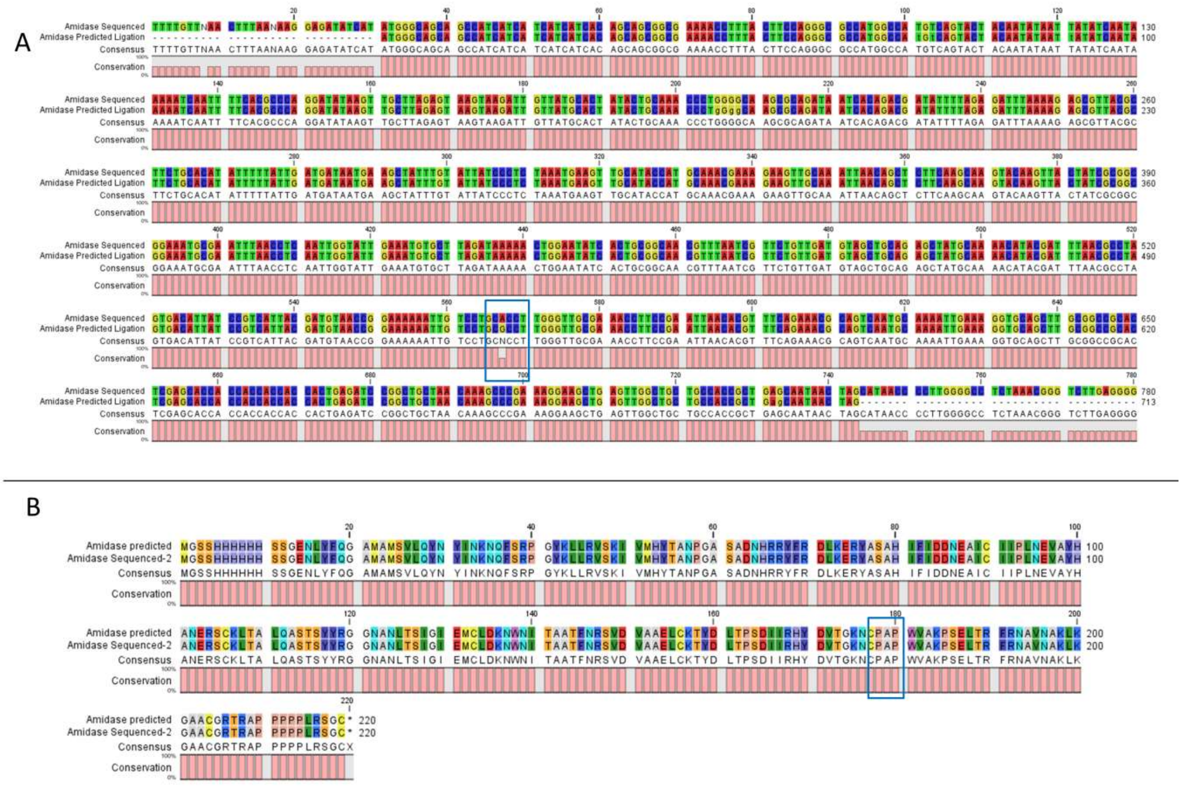

Figure S1. Local (A) DNA sequence alignment and (B) amino acid sequence alignment of predicted and cloned 293-amidase generated with CLC Main Workbench. A Single Nucleotide Polymorphism (SNP) is shown boxed in panel A showing a change from GCA to GCG in the cloned fragment of amidase-293.
