# Supplementary material for: Inhibition of L. monocytogenes Biofilm Formation by the Amidase Domain of the Phage vB_LmoS_293 Endolysin
Source: Viruses. 2019 Aug 6;11(8):722. doi: 10.3390/v11080722 (PMC6723838; doi:10.3390/v11080722)
Supplement: Supplementary file 1 [file viruses-11-00722-s001.zip › viruses-482321 final supplementary/Figure S2.pdf]

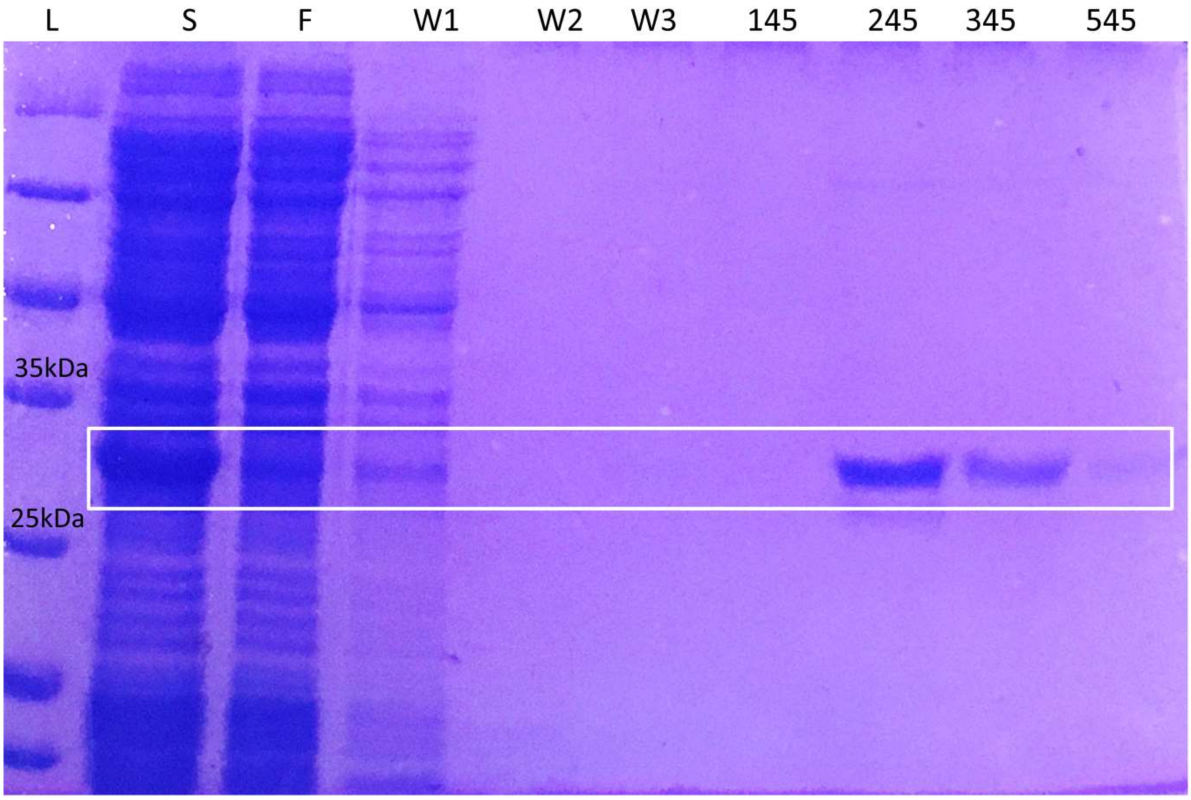

Figure S2. SDS-PAGE analysis of the 293-amidase purification showing the affinity chromatography fractions. The over-expressed protein band has been highlighted throughout the gel with a white rectangle and is clearly visible in the supernatant of the induced and lysed *E. coli* cells transformed with the pCri8A-293-amidase (S). After binding to the Ni-NTA resin, the band is less visible in the flow-through (F). L, ladder; S, supernatant of lysed cells; F, flow-through; W1 to W3, washing steps; 145 to 545, elution fractions with the values indicating the employed imidazole concentrations
