# Supplementary material for: Inhibition of L. monocytogenes Biofilm Formation by the Amidase Domain of the Phage vB_LmoS_293 Endolysin
Source: Viruses. 2019 Aug 6;11(8):722. doi: 10.3390/v11080722 (PMC6723838; doi:10.3390/v11080722)
Supplement: Supplementary file 1 [file viruses-11-00722-s001.zip › viruses-482321 final supplementary/Figure S3.pdf]

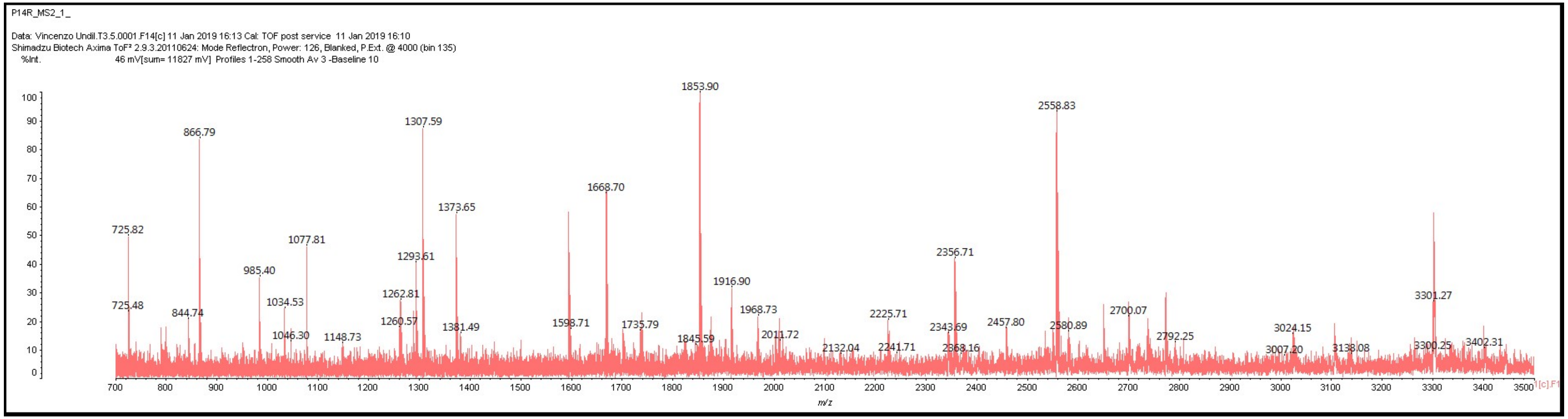

Figure S3. Mass spectrum of the 293-amidase digested with trypsin for identification by peptide mass fingerprint. On the horizontal axis are the masses and on the vertical axis is the abundance. Numbers on the peaks indicate the mass of the fragments obtained from the digestion with trypsin. A correspondence with the predicted sequence of the 293-amidase was found with the fragments: 1034.53, 1293.61, 1307.59, 1373.65, 1668.70, 1853.90 and 3301.27.
